# Association between blood viscosity and early neurological deterioration in lacunar infarction

**DOI:** 10.3389/fneur.2022.979073

**Published:** 2022-09-20

**Authors:** Hyungwoo Lee, JoonNyung Heo, Il Hyung Lee, Young Dae Kim, Hyo Suk Nam

**Affiliations:** ^1^Department of Neurology, Yonsei University College of Medicine, Seoul, South Korea; ^2^Integrative Research Center for Cerebrovascular and Cardiovascular Diseases, Yonsei University College of Medicine, Seoul, South Korea

**Keywords:** stroke, blood viscosity, prognosis, cerebral small vessel disease, disease progression

## Abstract

**Background:**

Understanding the factors related to early neurologic deterioration (END) is crucial in the management of patients with lacunar infarction. Blood viscosity is a significant factor for microvascular perfusion. We investigated the association between blood viscosity and occurrence of END in lacunar infarction.

**Methods:**

We included consecutive patients admitted for lacunar infarction within 72 h from symptoms onset. END was defined as an increase in the National Institute of Health Stroke Scale (NIHSS) score ≥2 within 24 h of admission. Viscosity was measured within 24 h of hospitalization with a scanning capillary tube viscometer. Viscosity measured at a shear rate of 300 s^−1^ was defined as systolic blood viscosity (SBV), whereas that measured at a shear rate of 5 s^−1^ as diastolic blood viscosity (DBV).

**Results:**

Of the 178 patients included (median age, 65.5; interquartile range [IQR], 56.0, 76.0], END occurred in 33 (18.5%). DBV was significantly higher in patients with END than those without END (13.3 mPa·s [IQR 11.8, 16.0] vs. 12.3 mPa·s [IQR11.0, 13.5]; *P* = 0.023). In the multivariate analysis, DBV was independently associated with the occurrence of END (odds ratio 1.17; 95% confidence interval 1.01–1.36; *P* = 0.043). Subgroup analysis showed no heterogeneity in the effect of viscosity on the occurrence of END.

**Conclusions:**

Blood viscosity at a low shear rate (DBV) was associated with the occurrence of END in patients with lacunar infarction. Blood rheology may be important in pathophysiology of END in patients with lacunar infarction.

## Introduction

Lacunar infarction, caused by single perforating artery occlusion, has shown more favorable outcomes than other stroke mechanisms (1, 2). However, 15–30% of patients with lacunar infarction suffer early neurologic deterioration (END), which is associated with poor prognosis (3–5). In that manner, END is a major concern in the treatment of patients with lacunar infarction. Factors associated with END include the location or shape of the ischemic lesion, initial symptom severity, presence of parent artery stenosis, and hypoperfusion (3, 4, 6–10). Of them, hypoperfusion may have therapeutic implication because it is modifiable.

Brain tissue perfusion may be altered by arterial pressure, vessel diameter, collateral status, temperature, or blood viscosity (11). Blood viscosity is a significant factor of microvascular perfusion (12). Previous studies have found that whole blood viscosity (WBV) was elevated in patients with ischemic stroke. Of note, the elevation was more significant in lacunar infarction than other stroke types (13). This might be related to the small diameter of the perforating artery and a low shear rate (13, 14). Under a low shear rate, which entails low flow or static flow, red blood cells (RBCs) tend to aggregate rapidly, thereby increasing blood viscosity (15–17). Elevated blood viscosity and flow resistance may alter tissue perfusion (16).

When considering the role of hypoperfusion in development of END, the association of blood viscosity with tissue hypoperfusion, and the elevated blood viscosity in patients with lacunar infarction, the blood viscosity may be associated with END in lacunar infarction. However, little is known about the relationship between END in lacunar infarction and blood viscosity. We hypothesized that elevated blood viscosity is a predictive marker for the occurrence of END in patients with lacunar infarction.

## Methods

### Study population

This was a retrospective, hospital-based observational study of patients who were registered to the Yonsei Stroke Cohort (Registry Number NCT03510312, www.clinicaltrials.gov). Consecutive patients admitted for acute ischemic stroke or transient ischemic attack were enrolled in the cohort. All patients underwent computerized tomography and/or magnetic resonance imaging with angiographic study. Demographic data, medical history, clinical manifestations, vascular risk factors, and comorbidities were evaluated. Systemic evaluation included 12-lead electrocardiography, chest radiography, standard blood tests, and lipid profiling during admission in the stroke unit. Most patients were admitted to the stroke unit and monitored continuously with electrocardiography. Transthoracic echocardiography and Holter monitoring were performed in selected patients. Transesophageal echocardiography was part of the standard evaluation except in patients with decreased consciousness, impending brain herniation, poor systemic conditions, inability to accept an esophageal transducer because of swallowing difficulty or tracheal intubation, and a lack of informed consent (18). From the cohort, patients with lacunar infarction who admitted within 72 h from the symptom onset were included in this study.

This study was approved by the institutional review board of Severance Hospital, Yonsei University Health System and the requirement for informed consent was waived due to the retrospective nature of the study.

### Clinical and laboratory data

Demographic data, vascular risk factors, and comorbidities, including hypertension, diabetes mellitus, dyslipidemia, coronary artery occlusive disease, peripheral artery occlusive disease, end-stage renal disease, cancer, previous stroke history, and premorbid modified Rankin Scale (mRS), were collected. Smoking status and alcohol consumption were also recorded. Men with >4 drinks at any single day or >14 drinks per week and women with >3 drinks at any single day or >7 drinks per week were defined as heavy alcoholics (19). Initial blood pressure was measured at the time of hospitalization.

Lacunar infarction was defined according to the Trial of ORG 10172 in Acute Stroke Treatment classification (20). a single lesion located in the basal ganglia, thalamus, internal capsule, corona radiata, and brain stem with maximal diameter <20 mm on diffusion-weighted MRI. The pial perforator infarction did not included. The etiologic classification of stroke, the lesion location, and the vascular territory of the lesion were determined during weekly conferences based on the consensus of stroke neurologists. Anterior lacunar infarction was defined as when the lesion was in the perforating artery territory of the anterior circulation (carotid, middle, and anterior cerebral arteries), and the remaining lesion types were categorized as posterior lacunar infarction. Nonsignificant relevant artery stenosis was defined as <50% stenosis of artery where perforator originate or any relevant artery stenosis of infarcted area on angiography, as determined by a radiologist. Poor premorbid functional status was defined as mRS 3–5 before index stroke. Initial neurologic severity was assessed on admission by a neurologist using the National Institute of Health Stroke Scale (NIHSS). An initial NIHSS 0–3 was defined as minor stroke (21). END was defined as any increase in NIHSS score ≥2 within 24 h of initial hospitalization (3).

Laboratory data collected included hemoglobin, hematocrit, white blood cell counts, erythrocyte sedimentation rate, glycated hemoglobin, creatinine, fibrinogen, d-dimer level, initial glucose, and blood viscosity.

### Blood viscosity

Whole blood was collected within 24 h after hospitalization and stored in ethylene-diamine-tetra-acetic acid tubes until analysis. Blood viscosity was assessed with a scanning capillary tube viscometer (Hemovister, Pharmode Inc., Seoul, Korea). The scanning capillary tube viscometer measures viscosity at different shear rates, as blood is a non-Newtonian fluid. In this study, viscosity measured at a shear rate of 300 s^−1^ was defined as systolic blood viscosity (SBV), whereas that measured at a shear rate of 5^−1^ was diastolic blood viscosity (DBV) (14, 22).

### Statistical analysis

Clinical and laboratory variables were compared between the END and non-END groups. The statistical significance of intergroup differences was assessed using the chi-squared test for categorical variables and independent two-sample *t*-test or Mann–Whitney U-test for continuous variables. Data were expressed as mean ± standard deviation or median (interquartile range [IQR]) for continuous variables and number (%) for categorical variables. The prevalence of END was compared at each quartile of blood viscosity. Univariable and multivariable logistic regression models were used to determine the significant factors for END. For the multivariable logistic analysis, variables with *P* < 0.05 in the univariate analysis were adjusted. According to the location of the ischemic lesion, blood viscosity was compared between the END and non-END groups. Subgroup analyses with logistic regression were also conducted for age, sex, premorbid disease, risk factors, initial neurological deficits, location of ischemic lesion, and presence of relevant artery stenosis. R version 4.0.5 (http://www.R-project.org; R Core Team, Vienna, Austria) was used to perform all statistical analyses, and two-tailed *P* < 0.05 was considered statistically significant.

## Results

A total of 1,168 consecutive patients were admitted from January 2020 to August 2021, of which 218 were diagnosed with lacunar infarction. After excluding 15 patients with insufficient data and 25 patients admitted after 72 h of symptoms onset, 178 patients were included for this study (Supplementary Figure 1).

Of 178 patients (median age, 65.5 years; IQR 56.0, 76.0), 107 (60.1%) were men. The median SBV was 3.9 mPa·s (IQR 3.7, 4.3), and the median DBV was 12.4 mPa·s (IQR 11.0, 13.5). Premorbid mRS was poor in 6 (3.4%) patients, whereas 27 (15.2%) patients had a previous stroke history. The median initial NIHSS score was 3 (IQR 1, 5). Eighty-five (47.8%) patients presented with anterior lacunar infarction, and 93 (52.2%) had posterior lacunar infarction. Nonsignificant relevant artery stenosis (<50%) was found in 67 (37.6%) patients (Table 1).

**Table 1:** Comparison of baseline characteristics on the presence of early neurologic deterioration.

	**END** **(*n =* 33)**	**No END** **(*n =* 145)**	**Total** **(*n =* 178)**	**P-value**
Age	70.0 [60.0;78.0]	65.0 [55.0;76.0]	65.5 [56.0;76.0]	0.139
Sex, Male	18 (54.5%)	89 (61.4%)	107 (60.1%)	0.598
Hypertension	27 (81.8%)	114 (78.6%)	141 (79.2%)	0.864
Diabetes	11 (33.3%)	54 (37.2%)	65 (36.5%)	0.825
Dyslipidemia	15 (45.5%)	67 (46.2%)	82 (46.1%)	1.000
Coronary artery occlusive disease	7 (21.2%)	15 (10.3%)	22 (12.4%)	0.156
Peripheral artery occlusive disease	3 (9.1%)	3 (2.1%)	6 (3.4%)	0.138
Previous stroke	8 (24.2%)	19 (13.1%)	27 (15.2%)	0.180
Poor premorbid mRS	2 (6.1%)	4 (2.8%)	6 (3.4%)	0.679
End-stage renal disease	1 (3.0%)	4 (2.8%)	5 (2.8%)	1.000
Cancer	2 (6.1%)	11 (7.6%)	13 (7.3%)	1.000
Current smoker	8 (24.2%)	40 (27.6%)	48 (27.0%)	0.862
Heavy alcoholics	3 (9.1%)	30 (20.7%)	33 (18.5%)	0.194
Hemoglobin (g/dL)	14.3 [13.6;14.9]	14.3 [13.0;15.3]	14.3 [13.1;15.2]	0.905
Hematocrit (%)	41.6 [40.3;43.0]	41.6 [38.6;44.6]	41.6 [38.9;44.4]	0.897
White blood cell count (10^3^/μL)	7.21 [5.84;9.07]	7.28 [6.06;8.71]	7.24 [6.05;8.73]	0.623
Creatinine (mg/dL)	0.8 [0.7; 1.0]	0.9 [0.8; 1.0]	0.9 [0.7;1.0]	0.327
Hgb A1c (%)	5.8 [5.4; 6.5]	5.9 [5.6; 6.6]	5.8 [5.5;6.6]	0.196
Initial glucose (mg/dL)	121.0 [105.0;132.0]	124.0 [106.0;161.0]	122 [106.0;157.0]	0.251
Erythrocyte sedimentation rate (mm/h)	6.0 [2.0;13.0]	6.0 [2.0;14.0]	6.0 [2.0;14.0]	0.801
Fibrinogen (mg/dL)	276.0 [260.0;307.0]	291.0 [251.0;329.0]	285.5 [252.0;329.0]	0.571
D-dimer (ng/mL)	149.0 [84.0;220.0]	125.0 [73.0;226.0]	126.0 [75.0;226.0]	0.480
Systolic BP	170.0 [144.0; 189.0]	164.0 [148.0; 184.0]	164.0 [148.0; 185.0]	0.712
Diastolic BP	84.0 [82.0; 99.0]	90.0 [80.0; 100.0]	89.0 [80.0; 100.0]	0.370
Mean BP	111.3 [103.3; 125.7]	116.3 [102.7; 126.0]	115.0 [103.3; 126.0]	0.661
Intravenous tPA	3 (9.1%)	7 (4.8%)	10 (5.6%)	0.588
Location of lacunar infarction				0.290
Anterior	19 (57.6%)	66 (45.5%)	85 (47.8%)	
Posterior	14 (42.4%)	79 (54.5%)	93 (52.2%)	
Initial NIHSS score	4.0 [3.0; 5.0]	3.0 [1.0; 5.0]	3.0 [1.0;5.0]	0.023
Nonsignificant relevant artery stenosis	19 (57.6%)	48 (33.1%)	67 (37.6%)	0.016
Onset to arrival (hour)	12.0 [7,1;15.5]	16.0 [7.7;30.0]	14.7 [7.7;28.2]	0.085
**Whole blood viscosity**				
SBV (mPa·s)	4.1 [3.7; 4.9]	3.9 [3.6; 4.2]	3.9 [3.7;4.3]	0.159
DBV (mPa·s)	13.3 [11.8;16.0]	12.3 [11.0;13.2]	12.4 [11.0;13.5]	0.010

Data are presented as median (interquartile range, [IQR]), or number (percentage). BP, blood pressure; NIHSS, National Institute of Health Stroke Scale; tPA, tissue plasminogen activator; SBV, systolic blood viscosity; DBV, diastolic blood viscosity.

END occurred in 33 (18.5%) patients and median increase in NIHSS after 24 h was 3 (IQR 2,3) (Supplementary Figure 2). Comparing with the patients without END, those with END had a higher initial NIHSS (4 [IQR 3, 5] vs. 3 [IQR 1, 3]; *P* = 0.003) and more frequent nonsignificant relevant artery stenosis (19 [57.6%] vs. 48 [33.1%]; *P* = 0.016). Other factors including hematocrit, fibrinogen, and erythrocyte sedimentation rate, which are associated with WBV, did not differ significantly between the groups (Table 1).

**Figure 1:**
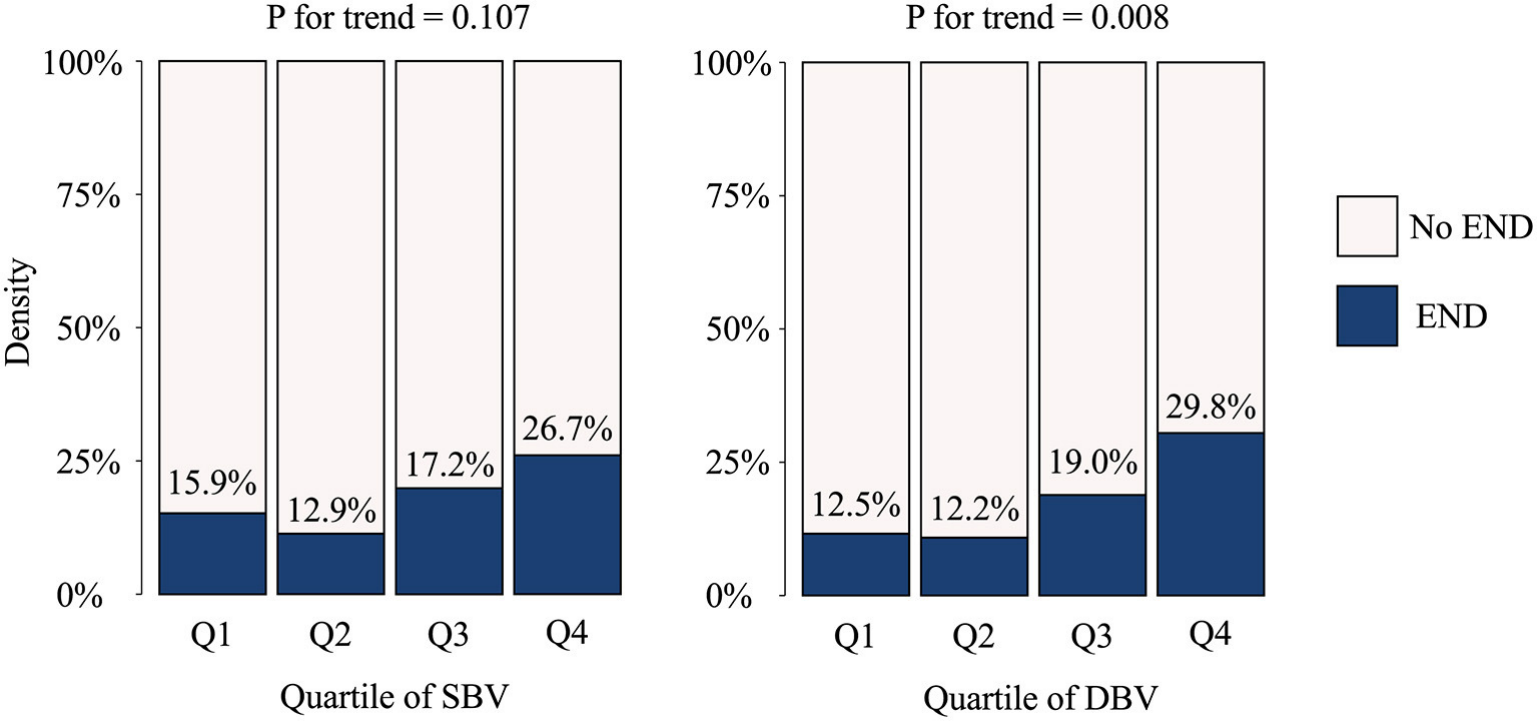
Occurrence of early neurologic deterioration per blood viscosity quartile. END, early neurologic deterioration; SBV, systolic blood viscosity; DBV, diastolic blood viscosity.

Patients with END had a higher DBV than those without END (13.3 mPa·s [IQR 11.8, 16.0] vs. 12.3 mPa·s [IQR 11.0, 13.5]; *P* = 0.023). However, no significant difference was found in SBV between the END and non-END groups (3.9 mPa·s [IQR 3.6, 4.2] vs. 4.1 mPa·s [IQR 3.7, 4.9]; *P* = 0.159) (Table 1). Linear by linear association showed that END frequently occurred in patients with higher quartiles for WBV, and the association was significant in DBV (*P* = 0.016 for quartiles), but not in quartiles for SBV) (*P* = 0.139) (Figure 1).

In multivariable logistic regression analysis, DBV was independently associated with the occurrence of END after adjusting for initial NIHSS score and presence of nonsignificant relevant artery stenosis (odds ratio 1.17; 95% confidence interval 1.01–1.36; *P* = 0.043) (Table 2).

**Table 2:** Univariable and multivariate logistic regression analyses for early neurologic deterioration.

	**Univariable analysis**		**Multivariable analysis**	
	**Odds ratio (95% CI)**	***P*** **-value**	**Odds ratio (95% CI)**	***P*** **-value**
Age	1.03 (0.99–1.06)	0.117		
Sex, Male	0.76 (0.35–1.62)	0.470		
Hypertension	1.22 (0.46–3.23)	0.683		
Diabetes	0.84 (0.38–1.87)	0.674		
Dyslipidemia	0.97 (0.45–2.07)	0.938		
Coronary artery occlusive disease	2.33 (0.87–6.29)	0.094		
Peripheral artery occlusive disease	4.73 (0.91–24.6)	0.065		
Previous stroke	2.12 (0.84–5.38)	0.113		
Poor premorbid mRS	2.27 (0.4–12.97)	0.355		
End stage renal disease	1.10 (0.12–10.19)	0.932		
Cancer	0.79 (0.17–3.73)	0.762		
Current smoker	0.38 (0.11–1.34)	0.134		
Heavy alcoholics	0.84 (0.35–2.02)	0.696		
Hemoglobin (g/dL)	1.01 (0.94–1.10)	0.737		
Hematocrit (%)	1.00(1.00–1.00)	0.713		
White blood cell count (10^3^/μL)	1.00 (0.99–1.01)	0.556		
Creatinine (mg/dL)	0.38 (0.11–1.34)	0.134		
Hgb A1c (%)	0.8 (0.57–1.14)	0.225		
Initial glucose (mg/dL)	1 (0.99–1.00)	0.259		
Erythrocyte sedimentation rate (mm/h)	1 (0.98–1.02)	0.935		
Fibrinogen (mg/dL)	1 (0.99–1)	0.539		
D-dimer (ng/mL)	1 (1–1)	0.820		
Systolic BP	1.00 (0.99–1.01)	0.903		
Diastolic BP	0.98 (0.96–1.01)	0.158		
Mean BP	0.99 (0.97–1.01)	0.408		
Intravenous tPA	1.97 (0.48–8.07)	0.345		
Location of lacunar infarction: Posterior	0.62 (0.29–1.32)	0.213		
Onset to arrival (hour)	0.98 (0.95 – 1.00)	0.065		
Initial NIHSS score	1.21 (1.00–1.47)	0.048	1.24 (1.01–1.51)	0.036
Nonsignificant relevant artery stenosis	2.74 (1.27–5.94)	0.010	2.25 (1.01–5.04)	0.048
DBV (mPa·s)	1.19 (1.03–1.38)	0.020	1.17 (1.01–1.36)	0.043
SBV (mPa·s)	1.54 (0.89–2.68)	0.125		

CI, confidence interval; BP, blood pressure; NIHSS, National Institute of Health Stroke Scale; tPA, tissue plasminogen activator; SBV, systolic blood viscosity; DBV, diastolic blood viscosity.

**Figure 2:**
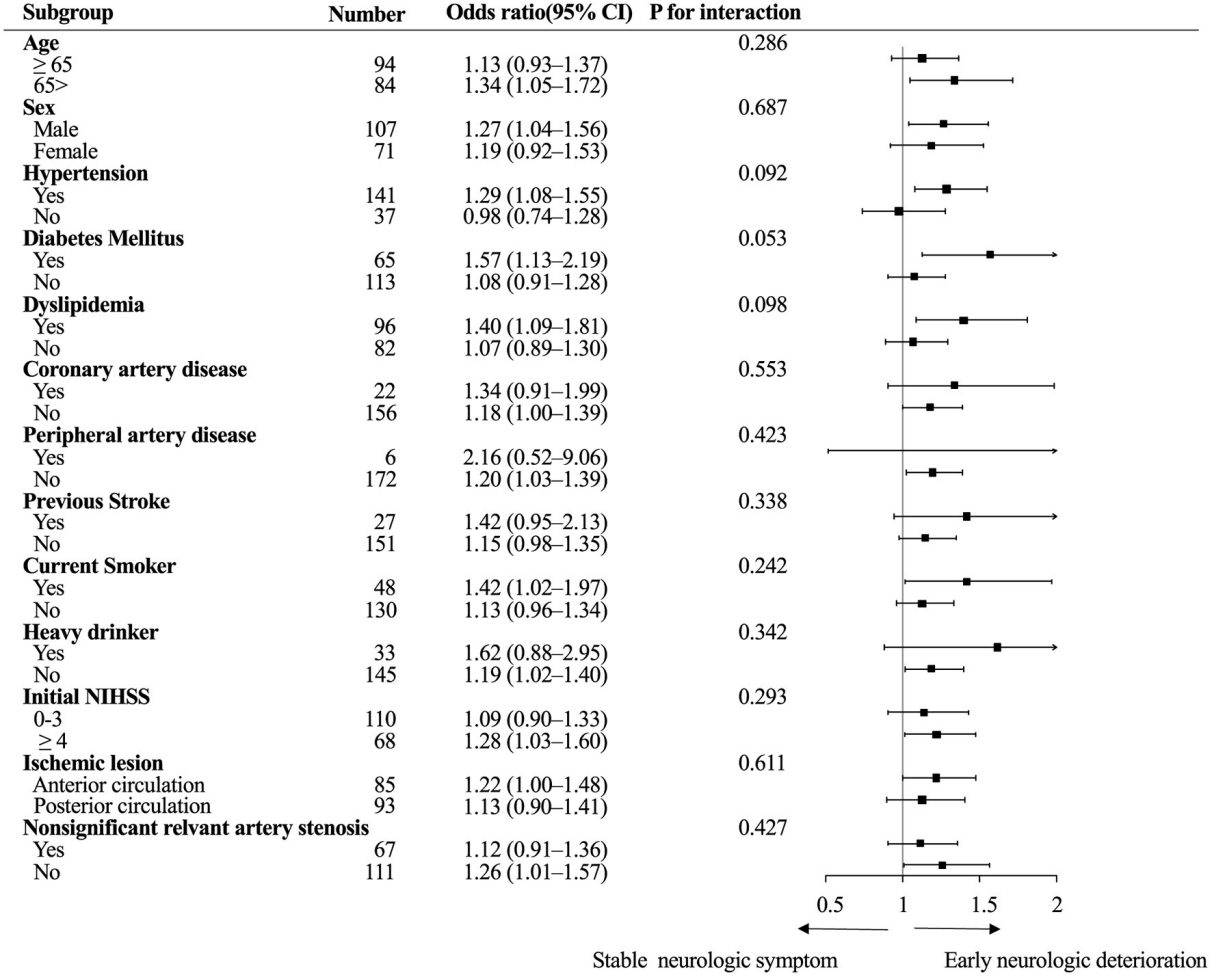
Subgroup analysis. Logistic regression analysis of the association between diastolic blood viscosity and early neurologic deterioration. CI, confidence interval; NIHSS, National Institute of Health Stroke Scale.

Subgroup analysis showed no heterogeneity in the effect of viscosity across the following variables in the occurrence of END. The direction of the effect of blood viscosity was associated with END across all strata (Figure 2).

**Figure 3:**
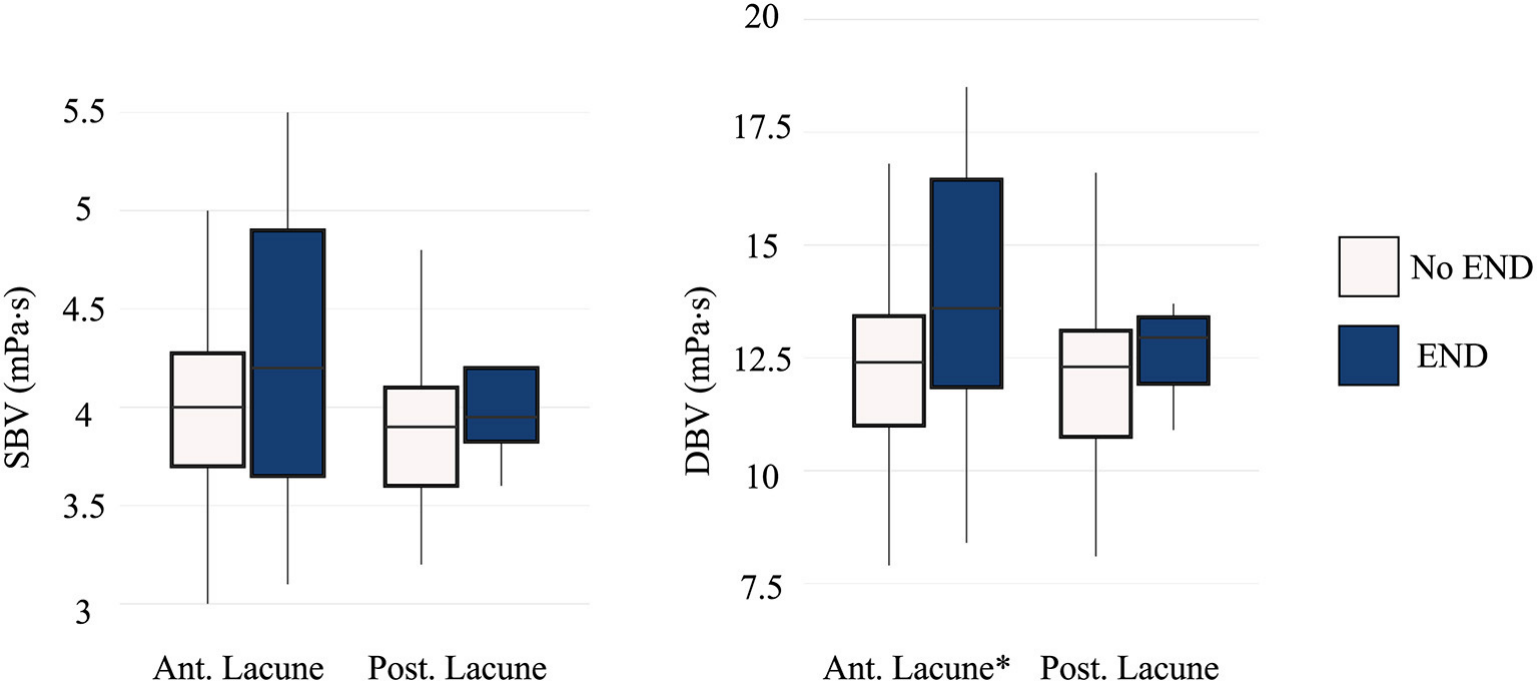
Comparison of blood viscosity between the END and non-END groups according to location of ischemic lesion. END, early neurologic deterioration; SBV, systolic blood viscosity; DBV, diastolic blood viscosity; Ant. Lacune, anterior lacunar infarction; Post. Lacune, posterior lacunar infarction. Patients with lacunar infarction in anterior circulation showed significant difference in END (*P* = 0.047). *p < 0.05.

Patients with lacunar infarction in anterior circulation who developed END showed higher DBV compared with those without END (13.6 mPa·s [IQR 11.8, 16.4] vs. 12.4 mPa·s [IQR 11.0, 13.5]; *P* = 0.047). However, we found no significant association between END and DBV in posterior lacunar infarction (Figure 3).

## Discussion

This study showed that DBV, defined as WBV in low shear rate, was significantly associated with the occurrence of END in patients with lacunar infarction. This association was still observed after adjusting for confounders, and the impact of viscosity on END was still observed in various subgroups.

Our study suggests that viscosity may play a role in the occurrence of END. Previous studies have shown that END in lacunar infarction was associated with perfusion delay on brain computerized tomography or magnetic resonance imaging performed before END (9, 10). Hypoperfusion is associated with arterial pressure, vessel diameter, collateral status, temperature, and blood viscosity (11). Elevated blood viscosity creates greater flow resistance to microvascular tissue perfusion, especially in pathological conditions wherein autoregulation fails, such as in acute ischemic stroke (23). Moreover, previous studies showed the relationship between END and hematocrit and acute phase reactant including fibrinogen (24, 25). The linkage between END and hematocrit and fibrinogen may be explained by blood viscosity. Elevation of hematocrit and fibrinogen were known to be associated with elevation of blood viscosity (26–28). This elevated blood viscosity could affect tissue perfusion and subsequently lead to END in patient with lacunar infarction. High blood pressure in the patients with elevated blood viscosity may also play a role. Previous study showed that blood viscosity is directly associated with blood pressure (29). Autonomic dysfunction of tissue perfusion in the patients with high blood pressure may have impact on END (30). In addition, underlying lipohyalinosis which is associated with hypertension (31) could contribute to END (32). Previous studies showed that initial blood pressure was associated with END (33, 34), although significant association was not obtained from this study.

We found that DBV was significantly associated with the occurrence of END, whereas SBV was not. A previous study reported that the blood viscosity at a low shear rate was more closely associated with lacunar infarction than that of high shear rate (14). In addition, blood viscosity at the low shear rate was significantly associated with silent brain infarction and subcortical vascular dementia caused by small vessel disease (35, 36). This shear rate-dependent relationship between END and blood viscosity seems reasonable because as the shear rate decreases, RBCs aggregate easily and form rouleaux, creating viscous drag in whole blood. Thus, viscosity increases exponentially as shear rate decreases (15). In consequence, DBV could more considerably impact microvascular perfusion than SBV in lacunar infarction.

In this study, differences in blood viscosity based on END status may be more evident in anterior lacunar infarction but no definite conclusion was obtained from this study. This may be related to the different diameters of vessels between anterior and posterior circulation. In an autopsy study on the normal human brain, the mean diameter of the middle cerebral artery perforators (469 μm) was larger than that of the basilar artery (393 μm) or the vertebral artery perforators (314 μm) (37). The diameter of the perforating artery strongly influences viscosity. In general, viscosity increases as diameter decreases. However, Fåhræus and Lindqvist showed that paradoxical effects can occur at diameters below 300 μm, where the apparent relative viscosity of blood decreases with decreasing diameter (38). Consequently, apparent relative viscosity decreases with decreasing vessel radius. This phenomenon is explained by the migration of RBCs toward the center of the vessel (thus moving faster), while a less viscous layer of plasma (ideally free of RBCs) forms close to the walls (39). According to Fåhræus–Lundquist effect, relatively smaller diameter of posterior perforating arteries might decrease the blood viscosity.

The relationship between blood viscosity and END in lacunar infarction may have therapeutic implication. Induced hypertension is possible treatment option for END (4, 40). As elevated blood viscosity increases flow resistance, hemodilution with intravenous fluid and induced hypertension therapy with phenylephrine might counteract the effects of elevated blood viscosity and improve tissue hypoperfusion (41–43). Further studies on these hemorheology factors may be necessary to identify targets for the treatment of END in lacunar infarction.

This study has some limitations. First, data were retrospectively collected from a single center; thus, a causal relationship cannot be drawn. Second, time from symptom onset to blood sampling varied among the enrolled patients. However, this study included only patients within 72 h from symptoms onset, and linear regression showed that the impact of time from symptoms onset to blood sampling on blood viscosity was minimal (*R*^2^ = 0.01, *P* = 0.008 for SBV; *R*^2^ = 0.02, *P* = 0.009 for DBV) (Supplementary Figure 3). Third, although hemodilution with intravenous fluid therapy could lower viscosity (23), the amount or type of fluid administered did not collected.

## Conclusion

This study showed that blood viscosity at a low shear rate was associated with the occurrence of END in patients with lacunar infarction. Our study suggests that decreasing blood viscosity may be helpful for preventing or treating END in lacunar infarction.

## Data availability statement

The original contributions presented in the study are included in the article/Supplementary material, further inquiries can be directed to the corresponding author/s.

## Ethics statement

The studies involving human participants were reviewed and approved by Institutional Review Board of Severance Hospital, Yonsei University Health System. Written informed consent for participation was not required for this study in accordance with the national legislation and the institutional requirements.

## Author contributions

HL: acquisition of data, analysis, and interpretation of data and writing of original draft. JH and IL: acquisition of data and interpretation of data. YK: interpretation of data and critical revision of the manuscript for intellectual content. HN: study concept and design, analysis, and interpretation of data and critical revision of the manuscript for intellectual content. All authors contributed to the article and approved the submitted version.

## Funding

This research was supported by a grant of Patient-Centered Clinical Research Coordinating Center (PACEN) funded by the Ministry of Health & Welfare, Republic of Korea (grant numbers: HI19C0481, HC19C0028).

## Conflict of interest

The authors declare that the research was conducted in the absence of any commercial or financial relationships that could be construed as a potential conflict of interest.

## Publisher's note

All claims expressed in this article are solely those of the authors and do not necessarily represent those of their affiliated organizations, or those of the publisher, the editors and the reviewers. Any product that may be evaluated in this article, or claim that may be made by its manufacturer, is not guaranteed or endorsed by the publisher.
