# Supplementary material for: Association between blood viscosity and early neurological deterioration in lacunar infarction
Source: Front Neurol. 2022 Sep 20;13:979073. doi: 10.3389/fneur.2022.979073 (PMC9530465; doi:10.3389/fneur.2022.979073)
Supplement: Supplementary file 1 [file Data_Sheet_1.docx]

SUPPLEMENTARY MATERIAL (online only)

**Association between blood viscosity and early neurological deterioration in lacunar infarction**

# Supplementary Figure 1. Flow chart of patient enrollment


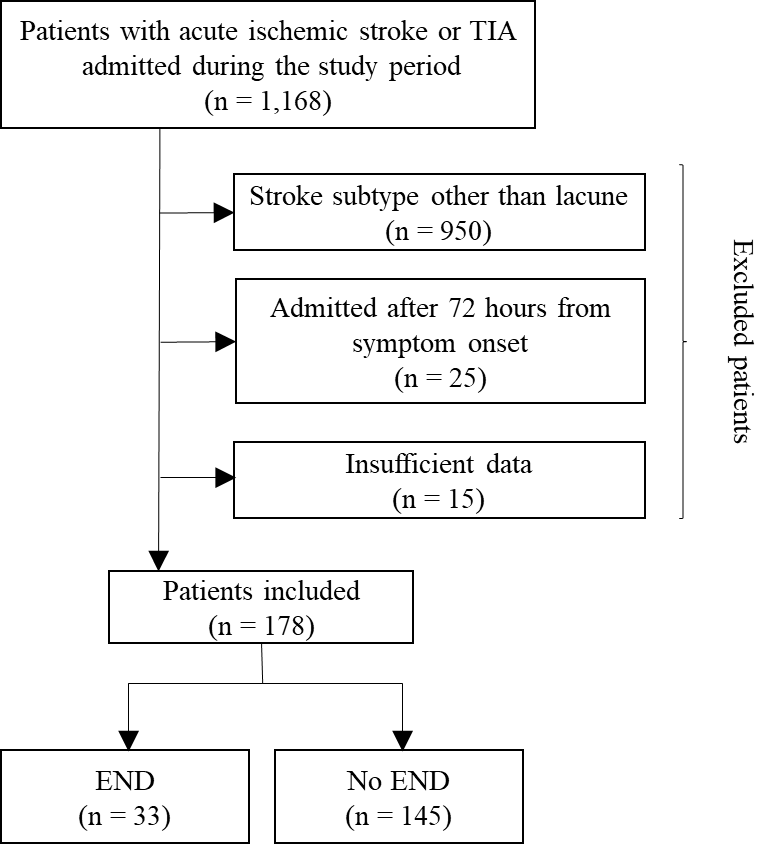


TIA, transient ischemic attack; END, early neurologic deterioration.

# Supplementary Figure 2. Serial National Institute of Health Stroke Scale score in patients with early neurologic deficit

END, early neurologic deterioration; NIHSS, National Institute of Health Stroke Scale; IQR, interquartile range.

# Supplementary Figure 3. Relationship between symptoms onset to sampling time and blood viscosity

SBV, systolic blood viscosity; DBV, diastolic blood viscosity.

**Supplementary Table 1.** Location of lacunar infarction and occurrence of END

|  | Basal  ganglia  (N=56) | Corona radiata  (N=9) | Internal capsule  (N=20) | Medulla  (N=8) | Mid brain  (N=3) | Pons  (N=48) | Thalamus  (N=34) | P value |
| --- | --- | --- | --- | --- | --- | --- | --- | --- |
| END |  |  |  |  |  |  |  | 0.073 |
| No | 46 (82.1%) | 8 (88.9%) | 12 (60.0%) | 7 (87.5%) | 3 (100%) | 37 (77.1%) | 32 (94.1%) |  |
| Yes | 10 (17.9%) | 1 (11.1%) | 8 (40.0%) | 1 (12.5%) | 0 (0.0%) | 11 (22.9%) | 2 (5.9%) |  |

END; early neurologic deficit.
